# Correspondence

**Published:** 1968-04

**Authors:** William H. Sweet

**Affiliations:** Chief Neurosurgical Service, Chairman, Committee on Management of the Unconscious Patient, Massachusetts General Hospital, Boston, Massachusetts 02114 USA


### CORRESPONDENCE

January 24, 1968

To The Editor,

Dear Sir :

The ability of physicians to maintain life for very long periods in the unconscious patient raises the question as to how long such skills should be deployed. As physicians we are eager to promote the recovery of everyone who can do so. In order to deprive no one of his chances on this score it is relevant to know the longest periods of coma which have been followed by useful survival.

A committee of the Massachusetts General Hospital is studying our own records and the world literature to determine pertinent features in all patients who, *despite coma for over 5 weeks,* have made a useful recovery. We think it is vital not to overlook any well documented patient in this category. We should be grateful if any reader of this journal would draw our attention to any case published under a title which is not indicative of survival after prolonged coma. We are also eager to receive accounts of such cases as yet unreported. A publication incorporating our own and others' data is planned.

We should be grateful if you would publish this letter in your journal either in a section for correspondence, as a special brief communication, or in any other fashion you see fit.

Sincerely yours,

WILLIAM H. SWEET, M.D., D.SC.
Chief Neurosurgical Service,
Chairman, Committee on Management of the Unconscious Patient,
Massachusetts General Hospital,
Boston, Massachusetts 02114 USA.

